# Pervasiveness and Frequency of Retinopathy of Prematurity in a Tertiary Care Centre and Its Correlation With Birth Weight and Gestational Age: A Retrospective Study

**DOI:** 10.7759/cureus.94804

**Published:** 2025-10-17

**Authors:** Supreetha Kumaresan, M Meera Devasena, Radha Annamalai, Adithya Tellakula, Venkat Meghana Bhimanadham, M Muthayya

**Affiliations:** 1 Ophthalmology, Sri Ramachandra Institute of Higher Education and Research, Chennai, IND; 2 Ophthalmology, Adithya Eye Clinic, Chennai, IND

**Keywords:** birth weight, gestational age, neonatal screening, prevalence, retinopathy of prematurity

## Abstract

Introduction

Retinopathy of prematurity (ROP) is a vasoproliferative retinal disorder affecting incompletely vascularized retina of preterm infants. It remains one of the leading preventable causes of childhood blindness worldwide. The incidence and severity of ROP vary with neonatal care standards, oxygen regulation, and demographic factors. Birth weight (BW) and gestational age (GA) are the most important predictors of disease, though other perinatal and systemic factors may contribute. In India, wide variation in reported prevalence underlines the importance of centre-specific data to guide screening protocols.

Aim

To determine the prevalence and severity of ROP in a tertiary-level hospital and to analyse its association with BW, GA, and retinal vascularization patterns.

Methods

This retrospective observational study was conducted at a tertiary hospital in South India from January 2022 to December 2024. Infants with GA ≤32 weeks and/or BW <2000 g, and those with GA 32-36 weeks with additional risk factors, were screened as per national guidelines. Data on maternal history, GA, BW, oxygen therapy, laboratory findings, retinal zone at first examination, and ROP stage were collected. Statistical analysis was performed using Microsoft Excel (Microsoft Corp., Redmond, WA) and IBM SPSS v29 (IBM Corp., Armonk, NY). Descriptive statistics and Pearson chi-square tests were applied, with p < 0.05 considered significant.

Results

A total of 375 infants were screened. A total of 50 infants (13.3%) had extremely low birth weight (ELBW), 171 (45.6%) had very low birth weight (VLBW), and 154 (41.1%) had low birth weight (LBW). Regarding gestational age (GA), 43 (11.5%) were extremely preterm, 156 (41.6%) were very preterm, and 176 (46.9%) were late preterm. A total of 12 (3.2%) had immature retinal vascularization in Zone I, 154 (41.1%) in Zone II, and 182 (48.5%) in Zone III, while 27 (7.2%) had a mature retina.

ROP was present in 19 infants (5.1%): Stage I in 11 (2.9%), Stage II in 8 (2.1%); plus disease was seen in one infant (0.3%). Retinal immaturity was significantly associated with lower BW (χ² = 104.387, df = 6, *p* = 0.0005) and earlier GA (χ² = 132.535, df = 6, *p* = 0.0005).

Conclusion

Prevalence of ROP in this tertiary hospital was 5.1%, with lower BW and earlier GA strongly associated with immature retinal vascularization. These findings underscore the importance of targeted screening and meticulous neonatal care to prevent sight-threatening ROP.

## Introduction

Retinopathy of prematurity (ROP) is a disorder of the developing retinal vasculature that occurs almost exclusively in preterm infants. It is characterized by abnormal retinal neovascularization and fibrovascular proliferation, which can progress to retinal detachment and irreversible blindness if left untreated [1]. First described in the 1940s as “retrolental fibroplasia,” the disease emerged as a consequence of improved survival of premature infants in high-income countries. Over the decades, advances in neonatal intensive care have altered the epidemiology of ROP, but it remains a major cause of preventable childhood blindness worldwide. The World Health Organization has identified ROP as one of the priority conditions under the “Vision 2020: The Right to Sight” initiative [2].

Epidemiology

The burden of ROP follows a “three epidemic” model. The first epidemic occurred in the mid-20th century in high-income countries when unrestricted oxygen supplementation to preterm infants led to high rates of blindness. Following the adoption of controlled oxygen use, the incidence declined dramatically. The second epidemic arose in the 1970s-80s with the survival of extremely premature infants due to advancements in neonatal care; these infants were at high risk despite careful oxygen titration. The third epidemic, currently ongoing in many middle-income countries, is driven by the survival of larger, more mature infants who develop ROP due to inconsistent oxygen monitoring, variable quality of NICU care, and lack of timely screening [3,4].

Risk factors

Birth weight (BW) and gestational age (GA) are consistently the most important predictors of ROP. The risk is highest in infants with BW <1000 g or GA <28 weeks; however, in countries such as India, China, and Brazil, cases are frequently observed in infants with higher BW and GA compared to high-income settings [5-7].

Other important risk modifiers include prolonged oxygen exposure, fluctuating oxygen saturation, sepsis, intraventricular hemorrhage, necrotizing enterocolitis, blood transfusions, apnea, and poor postnatal weight gain [8-10]. Of these, oxygen therapy remains the most important modifiable factor. Wide fluctuations in arterial oxygen saturation, especially in the early postnatal period, are strongly associated with severe ROP [11].

Pathophysiology

ROP develops in two overlapping phases. Phase I (22-30 weeks postmenstrual age) is characterized by delayed physiologic retinal vascular growth or vessel regression due to hyperoxia-induced downregulation of vascular endothelial growth factor (VEGF). In Phase II, as the metabolic demands of the retina increase and avascular retina persists, hypoxia stimulates excessive VEGF and insulin-like growth factor-1 (IGF-1) expression, resulting in abnormal neovascularization and, in severe cases, fibrovascular proliferation and tractional retinal detachment [9,12].

Early detection is critical, as timely treatment with laser photocoagulation or intravitreal anti-VEGF can prevent progression to blindness in most cases [9,13]. Screening guidelines vary by country. In India, the National ROP Screening Guidelines recommend screening all infants with GA ≤34 weeks or BW ≤2000 g, as well as selected larger or more mature infants with an unstable clinical course [14].

In India, reported ROP prevalence rates range from 20% to over 50% in screened populations, with 2%-10% developing treatment-requiring disease [4,15-17]. While large multicentric studies such as the “Indian Twin Cities ROP Study” have provided valuable prevalence and risk factor data [18], there remains a need for continuous local surveillance. Such centre-specific data help refine screening criteria, identify modifiable risk factors, and evaluate the impact of quality improvement initiatives in neonatal care.

Rationale for this study

While NICU care has advanced considerably, the population served includes a significant proportion of moderately preterm and low birth weight infants, many with comorbidities that could predispose them to ROP. Understanding the local prevalence and severity of ROP, and its correlation with BW, GA, and retinal vascularization patterns, is crucial for optimizing screening schedules and preventive strategies.

Hence, our objective is to determine the prevalence and severity of ROP in infants born at ≤32 weeks of gestation and/or with BW <2000 g (including selected infants 32-36 weeks with additional risk factors), and to evaluate correlations with BW and GA.

## Materials and methods

Study design and setting

This retrospective hospital-based observational study was conducted at Sri Ramachandra Institute of Higher Education and Research, Chennai, a tertiary care academic teaching hospital in South India. The study encompassed a three-year period from January 2022 through December 2024. The hospital provides comprehensive care to preterm and critically ill neonates from both urban and rural regions, thus offering an ideal platform for studying ROP prevalence and associated risk factors in a middle-income country context.

Ethics approval

The study protocol was reviewed and approved by the Institutional Ethics Committee. Given the retrospective nature of the study, the requirement for additional informed consent was waived. However, parental consent for routine ROP screening was obtained as part of standard clinical care. Data confidentiality was maintained at all times.

Inclusion and exclusion criteria

Infants admitted or visiting during the study period were eligible for inclusion if they met established criteria for ROP screening. Specifically, infants born at a gestational age (GA) of 32 weeks or less and/or with a birth weight (BW) under 2000 grams were included. Additionally, infants born between 32 and 36 weeks GA with recognized clinical risk factors, such as prolonged oxygen therapy or significant systemic illness, were also screened and included. Infants with congenital or genetic retinal abnormalities, ocular conditions that precluded adequate visualization of the fundus (e.g., dense cataract, corneal opacity), or incomplete screening records were excluded from the analysis to ensure data integrity and diagnostic accuracy.

Data collection

Data extraction was performed retrospectively using a standardized data collection proforma designed to capture relevant clinical, demographic, and ophthalmic information. Maternal history variables included antenatal steroid administration and documented maternal illnesses such as hypertension or diabetes, which can influence neonatal outcomes. Neonatal characteristics recorded comprised GA in completed weeks, BW in grams, sex, mode of delivery (vaginal or caesarean), and Apgar scores when available. They were categorised as follows

Gestational Age and Birthweight Categories

Gestational age was classified as extremely preterm (<28 weeks), very preterm (28-31 weeks), and late preterm (32-36 weeks), while birth weight was categorized as extremely low birth weight (<1000 g), very low birth weight (1000-1499 g), and low birth weight (1500-1999 g).

Screening protocol and ocular examination

Screening protocols were consistent with national and institutional guidelines. Anterior segment examination was performed using a torchlight to rule out anterior segment pathology.

Dilated fundus examination was conducted by an experienced retina specialist using binocular indirect ophthalmoscopy with a 20-diopter lens. Retinal vascularization was categorized based on the extent of retinal vessel growth into Zone I (central retina), Zone II (mid-peripheral retina), or Zone III (peripheral retina), or noted as mature if vessels had extended to the ora serrata. ROP was staged as per ICROP guidelines, with stages ranging from I (mild) to V (total retinal detachment) [12]. The presence of plus disease - dilated and tortuous posterior pole vessels indicating disease progression - was carefully assessed.

Infants with no ROP or immature retina were followed up at intervals defined by disease severity until full vascularization occurred. Treatment followed established protocols, including laser photocoagulation or intravitreal anti-VEGF therapy.

Sample size and statistical analysis

A sample of 375 infants who met the inclusion criteria and had complete screening data was analysed. Data were entered and cleaned in Microsoft Excel 2016 (Microsoft Corp., Redmond, WA) and imported into IBM SPSS Statistics version 29 for analysis (IBM Corp., Armonk, YA). Descriptive statistics, including frequencies, percentages, means, and standard deviations, summarized demographic and clinical variables.

Associations between retinal vascularization zones and BW/GA categories were evaluated using Pearson chi-square tests to detect statistically significant relationships. A p-value less than 0.05 was considered indicative of significance. The study’s retrospective nature and occasional missing data for oxygen metrics, transfusion details, and laboratory parameters limited the scope for multivariate regression analyses.

## Results

**Figure 1:**
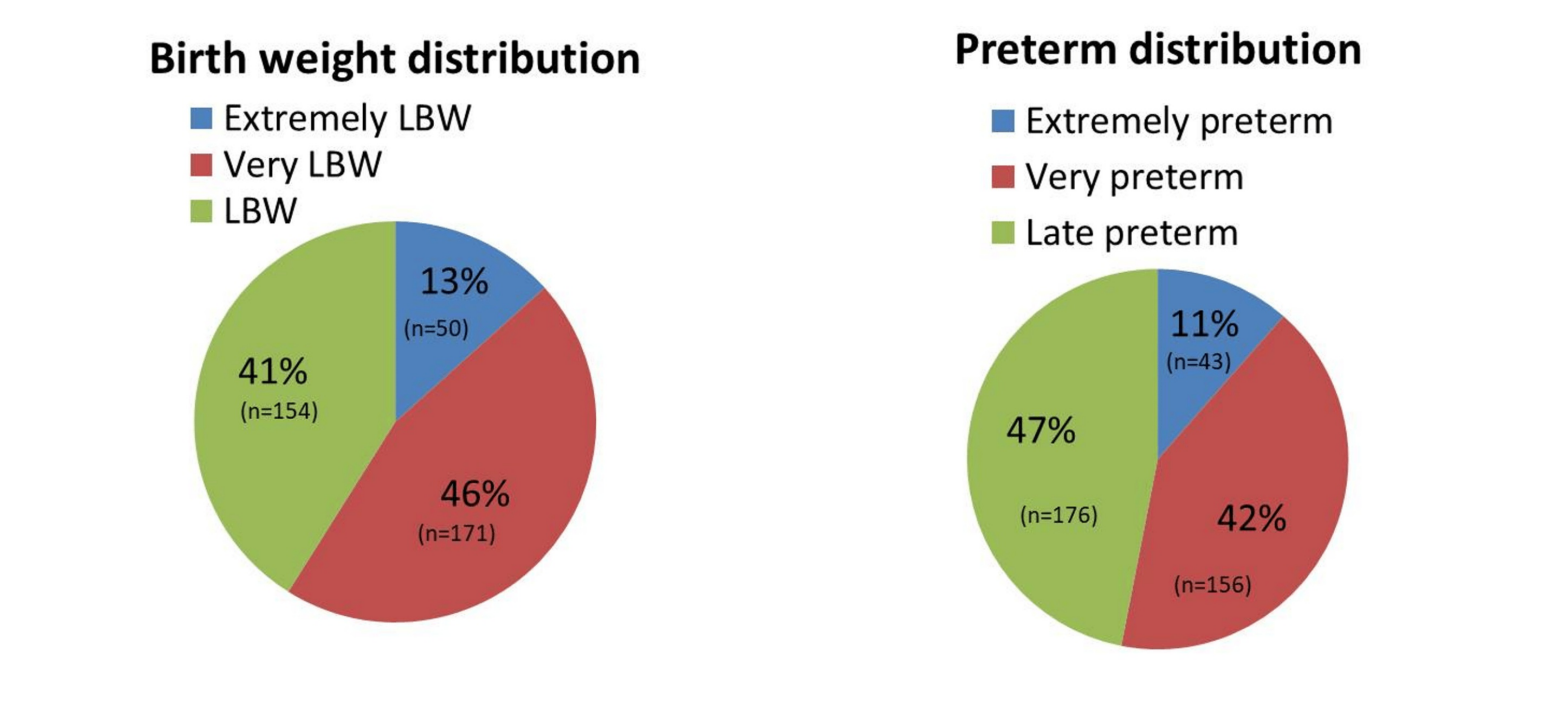
Cohort characteristics

A total of 375 neonates were screened for ROP. The majority were very low birth weight (VLBW, 45.6%) and late preterm (46.9%), with smaller proportions of extremely low birth weight (ELBW, 13.3%) and extremely preterm (11.5%) neonates (Figure 1).

Retinal vascularization patterns varied significantly across the cohort. Nearly half of the neonates (48.5%) exhibited immature vascularization in Zone III, while only 7.2% had fully mature retinas. Notably, immature Zone I vascularization, a marker of severely delayed retinal vascular growth, was observed in 3.2% of neonates (Table 1).

**Table 1:** Retinal vascularization patterns Zones classified as per The International Classification of Retinopathy of Prematurity (ICROP) [12]

Parameter	Frequency	Percentage (%)
Immature Zone I	12	3.2
Immature Zone II	154	41.1
Immature Zone III	182	48.5
Mature retina	27	7.2
Total	375	100

**Figure 2:**
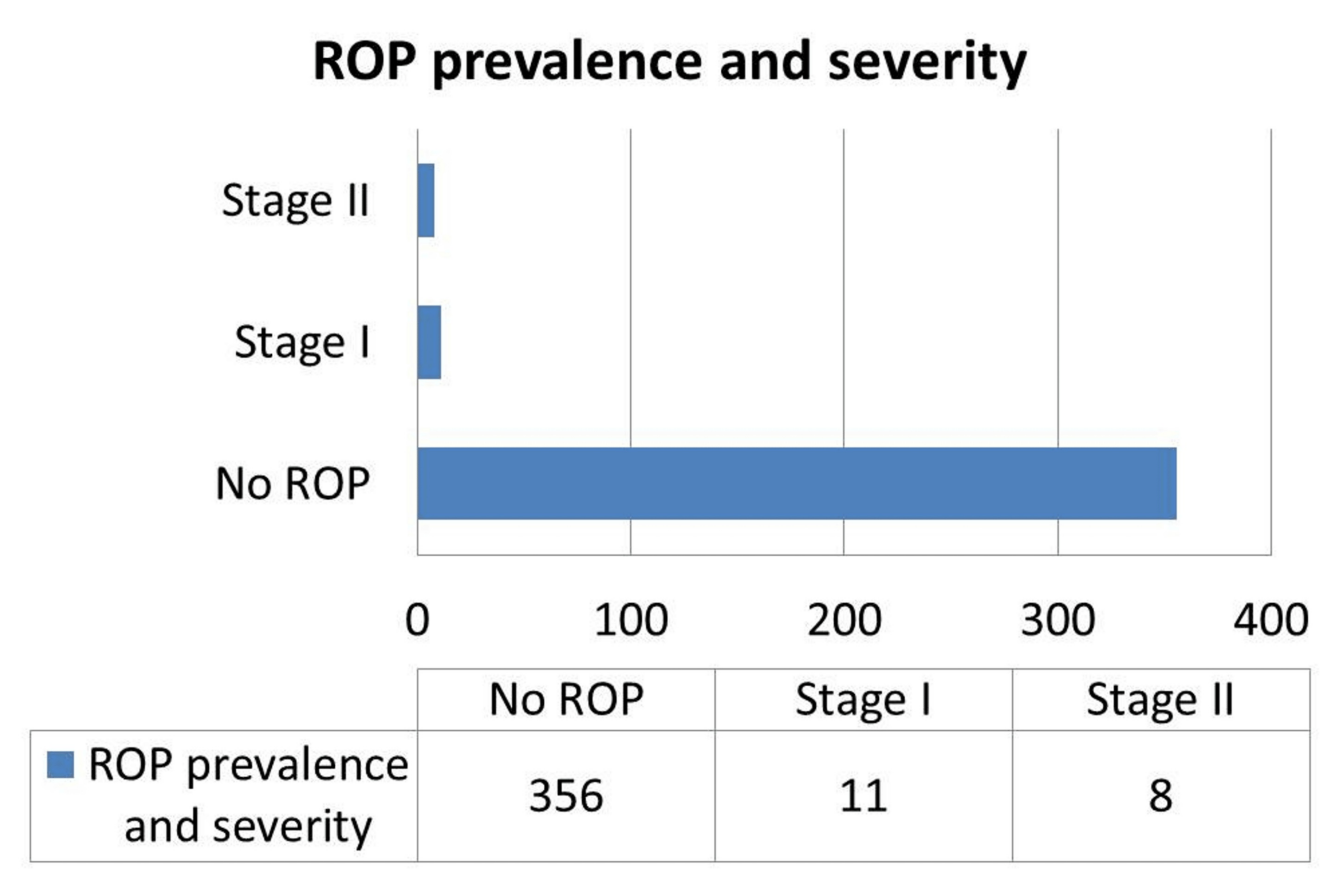
ROP distribution Staging as per The International Classification of Retinopathy of Prematurity (ICROP) [12]

ROP was diagnosed in 5.1% of neonates, predominantly Stage I (2.9%) and Stage II (2.1%). No cases of advanced ROP (Stage III or higher) were observed during the study period. Plus disease was rare, present in only one of the neonates (Figure 2).

**Figure 3:**
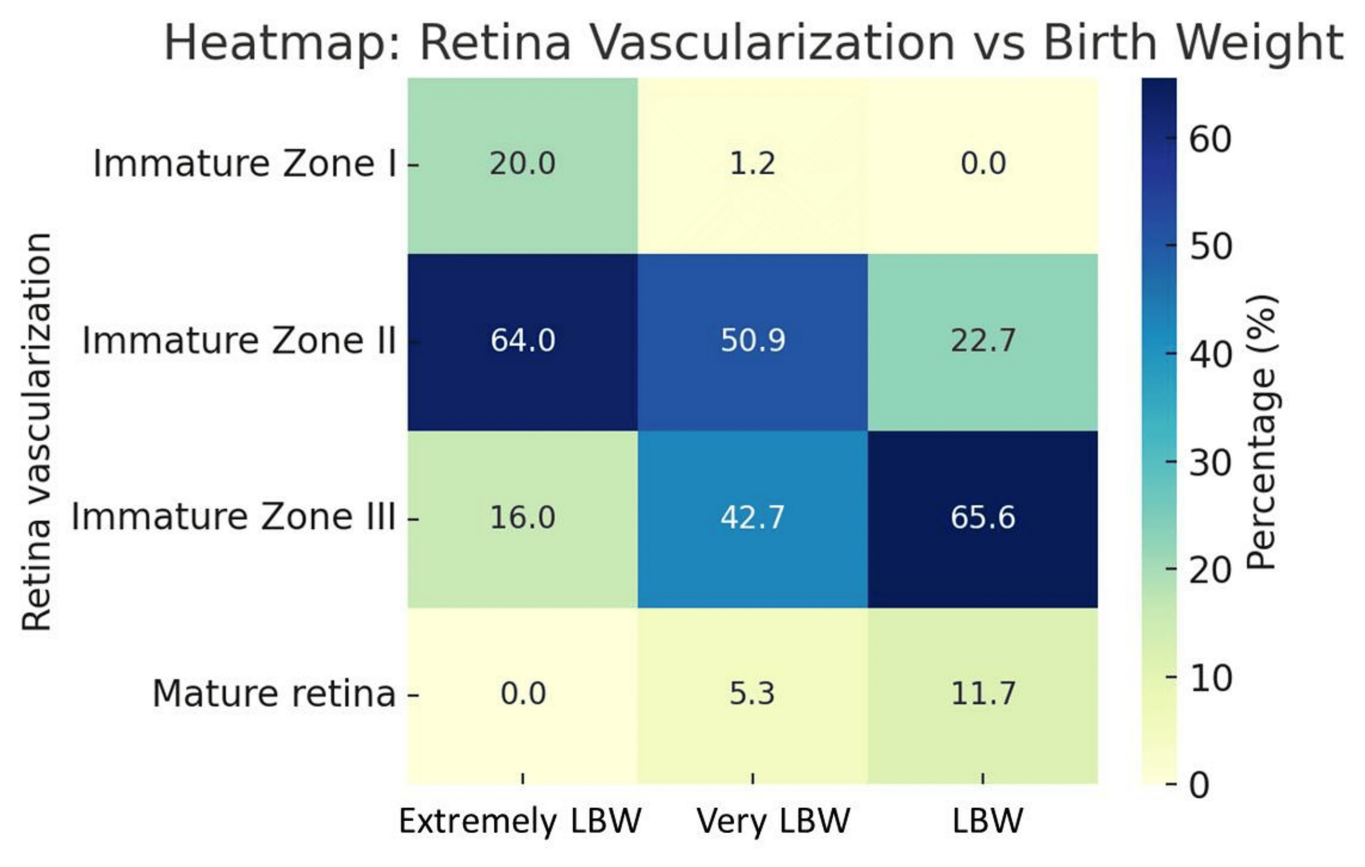
Retinal vascularization vs birth weight heatmap Zones classified as per The International Classification of Retinopathy of Prematurity (ICROP) [12]

A strong correlation was found between the birth weight and retinal vascularization (p = 0.0005). ELBW neonates showed a notably higher prevalence of immature Zone I vascularization (20.0%) compared to VLBW (1.2%) and LBW (0%). Conversely, mature retinas were most common among LBW neonates (11.7%), suggesting accelerated retinal maturation with increasing birth weight (Figure 3).

**Figure 4:**
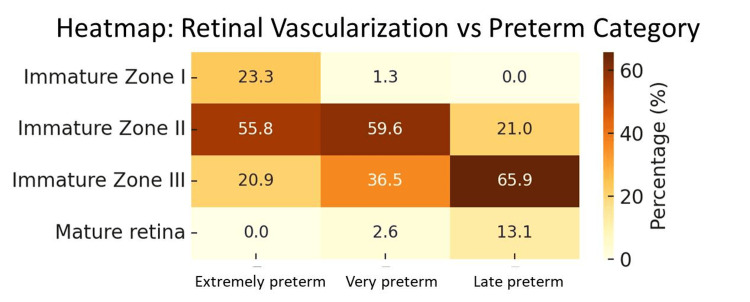
Retinal vascularization vs gestational age heatmap Zones classified as per The International Classification of Retinopathy of Prematurity (ICROP) [12]

Similarly, gestational age was significantly associated with retinal vascularization (p = 0.0005). Extremely preterm neonates had the highest proportion of immature Zone I retina (23.3%), while late preterm neonates more frequently had mature retinas (13.1%). This highlights delayed retinal vascular development in neonates born at earlier gestational ages. (Figure 4).

Lower birth weight and earlier gestational age strongly predict delayed retinal vascularization, increasing vulnerability to ROP. The absence of advanced-stage ROP and low plus disease prevalence may reflect effective neonatal care or early screening.

## Discussion

In this study of 375 infants, ROP was identified in 5.1% of screened infants and was limited to early-stage disease (Stages I and II). Retinal vascular immaturity, particularly within Zones I and II, demonstrated a statistically significant association with both lower birth weight (BW) and earlier gestational age (GA) (p < 0.001). These findings are in alignment with well-established literature identifying BW and GA as the strongest and most consistent predictors of ROP risk and progression, underscoring their pivotal role in retinal vascular development and disease pathogenesis [1,3,5,6,9].

The ROP prevalence observed in our study is considerably lower than that reported in many Indian tertiary care centres, often between 14% and 45%, reflecting a heterogeneous population and differing standards of neonatal care [4,15,16,18]. This variability likely arises from a confluence of factors, including the level and quality of neonatal intensive care, oxygen supplementation practices, screening policies, and patient selection criteria. For example, units with more liberal oxygen administration or less stringent saturation monitoring are known to experience higher rates of ROP, particularly severe disease [3,4,10,11]. Moreover, centres with broader screening criteria or those serving populations with a greater proportion of extremely preterm or very low birth weight infants often report higher prevalence and severity of ROP [15,16].

In our setting, the relatively low prevalence of ROP and the absence of advanced stages may indicate effective oxygen stewardship, timely and systematic screening, and improved neonatal management protocols. The adherence to national guidelines for screening and the consistent involvement of retina specialists in examinations could also enhance early detection and appropriate monitoring, preventing progression to threshold disease [10,11,13].

The absence of treatment requiring ROP does not exclude the possibility of late progression, especially in infants who may have been lost to follow-up after early examinations. Literature indicates that even initially mild ROP can progress to more severe forms, particularly in infants with ongoing systemic instability such as sepsis, fluctuating oxygen saturation, blood transfusions, and inadequate postnatal weight gain [6,9,14,19]. These risk factors, well-documented in various populations, highlight the need for vigilant longitudinal follow-up until full retinal vascularization is confirmed [13].

The incorporation of advanced screening modalities such as teleophthalmology and wide-field digital retinal imaging holds promise to extend screening coverage and improve early detection, particularly in resource-limited or geographically remote settings [13,14,18,20]. The success of tele-imaging initiatives in India exemplifies how technology-enabled care models can address disparities in access to specialized ophthalmic services.

This study also highlights the critical need for enhanced documentation of risk variables beyond BW and GA. While these primary predictors were reliably recorded and strongly correlated with retinal immaturity in our study, retrospective data often lacked granular information on oxygen exposure parameters, transfusion specifics, sepsis episodes, and postnatal growth trajectories. The absence of these detailed metrics limits the ability to develop locally relevant multivariate risk prediction models or tailor screening protocols to specific neonatal populations [5,7,9,19,21]. Prospective multicentre studies are needed to develop validated risk-prediction models that integrate clinical and systemic factors. These will inform decisions regarding potential expansion or refinement of screening criteria, optimizing resource use while ensuring at-risk infants are identified and treated promptly. Such efforts will be crucial to reducing preventable childhood blindness caused by ROP in India and similar middle-income countries.

Strengths of our study include a relatively large cohort and consistent retinal examinations performed by experienced specialists, minimizing inter-observer variability in disease classification. However, the retrospective and single-centre design restricts the generalizability of findings.

## Conclusions

In this retrospective study conducted at a tertiary care NICU, the prevalence of ROP was 5.1%, with all cases limited to early stages (Stages I and II). Retinal vascular immaturity showed a strong and significant correlation with lower BW and earlier GA (p < 0.001), reaffirming these as the primary risk factors for ROP development. These results are consistent with existing literature emphasizing prematurity and low BW as key predictors of ROP risk.

This low prevalence of ROP and lack of advanced disease suggest that effective neonatal care, particularly careful oxygen management and timely screening, plays a key role. Adherence to established screening protocols and high standards of neonatal care is vital to prevent severe ROP. Consistent documentation of modifiable risk factors, including oxygen use and transfusions, would strengthen risk assessment and help develop more precise, locally tailored screening strategies.
